# PhyDesign: an online application for profiling phylogenetic informativeness

**DOI:** 10.1186/1471-2148-11-152

**Published:** 2011-05-31

**Authors:** Francesc López-Giráldez, Jeffrey P Townsend

**Affiliations:** 1 Department of Ecology and Evolutionary Biology, Yale University, New Haven, Connecticut, USA

## Abstract

**Background:**

The rapid increase in number of sequenced genomes for species across of the tree of life is revealing a diverse suite of orthologous genes that could potentially be employed to inform molecular phylogenetic studies that encompass broader taxonomic sampling. Optimal usage of this diversity of loci requires user-friendly tools to facilitate widespread cost-effective locus prioritization for phylogenetic sampling. The Townsend (2007) phylogenetic informativeness provides a unique empirical metric for guiding marker selection. However, no software or automated methodology to evaluate sequence alignments and estimate the phylogenetic informativeness metric has been available.

**Results:**

Here, we present PhyDesign, a platform-independent online application that implements the Townsend (2007) phylogenetic informativeness analysis, providing a quantitative prediction of the utility of loci to solve specific phylogenetic questions. An easy-to-use interface facilitates uploading of alignments and ultrametric trees to calculate and depict profiles of informativeness over specified time ranges, and provides rankings of locus prioritization for epochs of interest.

**Conclusions:**

By providing these profiles, PhyDesign facilitates locus prioritization increasing the efficiency of sequencing for phylogenetic purposes compared to traditional studies with more laborious and low capacity screening methods, as well as increasing the accuracy of phylogenetic studies. Together with a manual and sample files, the application is freely accessible at http://phydesign.townsend.yale.edu.

## Background

Due to advances in sequencing technologies and decreasing costs, the number of genomes sequenced for species across the tree of life is increasing dramatically. Tools and databases for selecting single-copy orthologous loci [1,2] and designing successful primers for them [3,4] are available. However, orthology assessments for multiple genomes can provide thousands of candidate loci to sequence, and yet only a few of those have been commonly used as markers for phylogenetic studies [5]. To address the challenge of locus selection for sequencing in designing a phylogenetic study, Townsend [6] proposed a metric that provides a quantitative prediction of phylogenetic signal across historical times. Based on estimates of rates across sites, the phylogenetic informativeness metric facilitates prioritization of loci even when the taxa of interest have never been sequenced for a given locus. To estimate phylogenetic informativeness, prior data on the molecular evolutionary pattern of a locus is required. This prior information may be derived from three potential sources: 1) preliminary data on the candidate loci from a well-studied subset of the taxa of interest; 2) data on the candidate loci from a well-studied sister clade; or 3) comparative genomic data from sequenced genomes within and/or outside the clade of interest. Using this information for a number of classic and recent data sets for different time scales, the method successfully recapitulates the qualitative utility of loci [6-10]. However, no software or automated methodology to apply the phylogenetic informativeness metric has been available.

To facilitate wider application of phylogenetic informativeness analysis, we have developed the online application PhyDesign. PhyDesign features:

• Online accessibility, platform independence, and immediate access to software updates.

• User-friendly graphical interface with rich process feedback.

• Multiple locus analyses of amino acid and DNA alignments and multiple epoch integration.

• High quality graphical outputs in SVG format easily edited for final publication.

• Spreadsheet outputs including site rate estimates and phylogenetic informativeness values in rank-order of priority for the epochs of interest, as well as tables to reproduce the profiles for further processing.

PhyDesign is freely accessible online at http://phydesign.townsend.yale.edu. A manual and sample files to be used with the application, as well as a FAQ section, can be also found at the site. In addition, the source code and a Perl module to calculate the profiles are available for download.

## Implementation

To estimate phylogenetic informativeness profiles [6], the PhyDesign application consists of 3 components:

1. A form to upload information and choose an application to calculate the evolutionary rates for each alignment site.

2. A table listing the evolutionary rate results.

3. A graphical interface to plot the phylogenetic profiles and calculate integration values.

On the client-side, these components of PhyDesign are coded in JavaScript/AJAX using jQuery libraries, facilitating dynamic communication between the server and the user without page reloading. Online input undergoes validation to detect possible errors before engaging the next step in the process. To communicate validation success or failure and its causes, unobtrusive temporary message boxes are featured. In addition, the web site uses modal windows to provide optional advanced settings. On the server-side, PhyDesign is implemented as a collection of Perl scripts and modules, integrated with third-party software used to calculate the evolutionary rates for each site in the alignment.

To run, PhyDesign requires at minimum an internet browser equipped with JavaScript, and SVG for the graphical output. The latest versions of major modern browsers have native support for JavaScript and for rendering SVG markup directly. Internet Explorer 8 and older versions require a plug-in to render SVG content. SVG format, as vector graphics format, is resolution-independent and easy to edit for final publication with common vector graphic editors such as Adobe Illustrator (commercially available) and Inkscape (open source). The online application has been tested as rendered by Internet Explorer, Firefox, Chrome and Safari. Although graphical representations of phylogenetic informativeness profiles are typically best kept to lower numbers of loci for purposes of display, PhyDesign was able to profile and produce quantitative results for 2000 loci of a length of 1000 bp each. For a higher volume of data, a Perl module to calculate the profiles is available for download.

### Input form

To obtain the site rate distributions for each locus, two data entries are needed: (1) an alignment of loci of interest, pruned to contain a set of taxa for which the tree topology is fairly well known, and (2) an ultrametric tree for those taxa. The ultrametric tree can be a chronogram (an ultrametric tree with branch lengths proportional to time). Alternatively, branch lengths can be in unspecified molecular evolutionary units. PhyDesign reads amino acid and DNA alignments in NEXUS, FASTA, and Phylip formats, and trees in NEXUS and Newick formats. Formats are carefully checked via custom and BioPerl modules [11]. Data partitioning is permitted via NEXUS format. Once files are uploaded and checked, PhyDesign provides an interface for selecting and parameterizing any one of several third-party programs that estimate the site rates - HyPhy [12], DNArates [Olsen, unpublished] and Rate4site [13]. DNArates and Rate4site are called by the application "as is"; a *de novo *HyPhy script was implemented to estimate rates under the full diversity of time-reversible models. Due to the ability to specify of the evolutionary model and its parameters, HyPhy is the recommended and default option for DNA sequences. For amino acid sequences, Rate4site is available. To facilitate extensive analyses of large datasets, a link to the rates results is sent by email. A site rate file with the proper formatting will be generated after analysis of the alignments and the corresponding ultrametric tree. Supplied alignments are deleted from the server immediately after the site rates are calculated, and the site rate results are stored in the server for 24 hours. During this time, results can only be accessed by the link submitted. It is convenient, then, to save the site rate file for future use. Alternatively, if the rate distribution for each locus is known, PhyDesign also accepts direct supply of these rates to obtain the phylogenetic informativeness profiles.

## Results and Discussion

### Output of rate vectors

The first result provided features a table with basic information about each locus analyzed, including the program used to analyze the rates, locus length, the number of sites for which a substitution rate could be calculated, and the number of faulty sites for which this calculation was not possible. Two files are offered as downloads: (1) a compressed file containing individual rate files for each locus, and (2) a single file containing rate site vectors for all loci. The latter can be downloaded for future uploading in the rate vectors form, eliminating the need to repeat rate calculations. Partitions to be profiled and the colors for representing them may then be specified interactively.

### Profiles and area calculations

**Figure 1:**
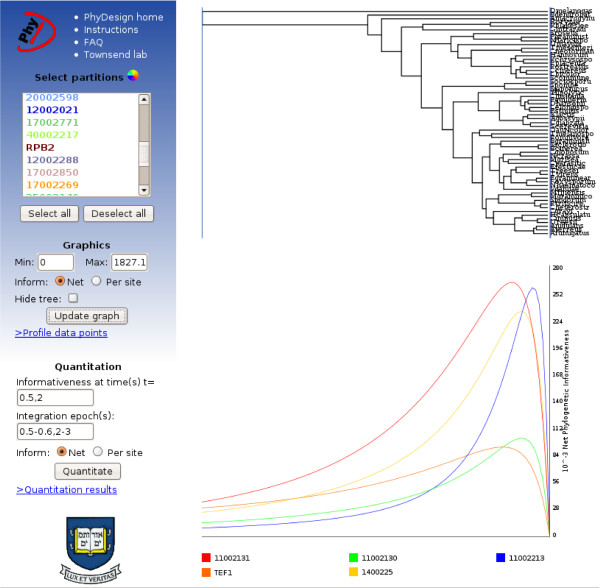
**Screenshot of PhyDesign**. Phylogenetic informativeness profiles are shown for five loci, aligned to a corresponding chronogram. The side bar is the interface through which the user can select loci and colors, adjust the range of time values, integrate over different epochs, and download the results.

The second result provided is a graphical representation of phylogenetic informativeness and calculations of quantitative measures of informativeness. Two graphs are shown in the main section: the ultrametric tree and, aligned to it, the phylogenetic informativeness profiles (Figure 1).

These graphs can be readily downloaded in manipulable SVG format as displayed. In addition, a downloadable spreadsheet file with the profile data points is provided in the left panel, so that profiles can be replotted or reanalyzed with diverse software. The left panel facilitates further customization of the plots, including selection of loci to display, colors of profiles, adjustment of the range of time values, integration over different epochs, and downloading of the results. Integrating phylogenetic informativeness over specific epochs provides a metric for ranking loci. Integration values will be largest for the loci that have the highest probability of substitution in the given epoch that will not be obscured by subsequent evolution. Note, however, that phylogenetic informativeness plots display predicted signal and do not account for phylogenetic noise (homoplasy) caused by convergence or parallelism in divergent lineages. Thus, quantitative results should be thoughtfully considered in light of homoplasy that is likely to arise, significantly diminishing utility during epochs deeper than the peak of informativeness for a given profile. All informativeness values can be calculated on a net or per site basis. While the net phylogenetic informativeness quantifies signal as a whole, it is more subject to phylogenetic noise [14,15] than is phylogenetic informativeness per site, which maximizes both informativeness and cost-effectiveness, and more effectively minimizes noise. It also of conceptual interest to characterize the phylogenetic informativeness per site to compare relative power of genes without the confounding influence of gene length. A combination of shorter genes with a sequencing effort equal to that of a longer gene can lead to better results.

## Conclusions

By providing these profiles, PhyDesign facilitates locus prioritization, increasing the efficiency of sequencing for phylogenetic purposes compared to traditional studies with more laborious and low capacity screening methods, as well as increasing the accuracy of phylogenetic studies. Future website implementations will include the latest theoretical advances developed in our research group, expanding the current phylogenetic informativeness methodology to quantify the effects of parallelism and convergence, as well as quantifying the utility of taxon addition [16].

## Availability and requirements

Project name: PhyDesign

Project home page: http://phydesign.townsend.yale.edu

Operating system(s): Platform independent

Programming language: JavaScript and Perl

Other requirements: Internet browser with JavaScript and SVG for the graphical outputs (supported by all major browsers).

License: The tool is available online free of charge, and code is available under a Creative Commons Attribution-ShareAlike 3.0 Unported License.

Any restrictions to use by non-academics: None

## Authors' contributions

FL-G and JPT conceived and designed the implementation. FL-G developed the online interface and server Perl scripts. FL-G wrote the manuscript. All authors read, edited, and approved the final manuscript.
